# Supplementary material for: Biopsychosocial approach to understanding predictors of depressive symptoms among men who have sex with men living with HIV in Selangor, Malaysia: A mixed methods study protocol
Source: PLoS One. 2023 Jun 2;18(6):e0286816. doi: 10.1371/journal.pone.0286816 (PMC10237489; doi:10.1371/journal.pone.0286816)
Supplement: S1 File — (DOCX) [file pone.0286816.s001.docx]

Interview Protocol (English version)

Ethic ref: NMRR ID-21-02210-MIT


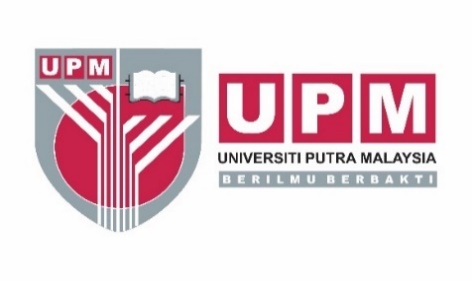


**UNIVERSITI PUTRA MALAYSIA COMMUNITY HEALTH MEDICINE DEPARTMENT**

INTERVIEW PROTOCOL

(In-depth interview)

Time:

Date:

Place:

Interviewer:

Respondent code:

**Interview Guide – Depressive Symptoms**

Note: Please read or paraphrase the interview script provided below.

Introduction:

Assalamualaikum/good morning/evening. I’m …………………………..from Universiti Putra Malaysia. I’m currently conducting a study for my Doctor of Public Health (DrPH) project regarding depressive symptoms among HIV-positive men. We appreciate your participation in this study.

Today’s purpose of this interview is to find out from you and other patients in this clinic your experience with feeling sadness or depressive symptoms and understand the associated factors.

The meeting will last approximately sixty minutes. I will utilize a recording device to capture our discussion to make a transcript of the conversation. To secure your identity, please refrain from using your name or the names of any people you know.

Are you ready to begin the interview? Do you have any questions before we start?

**General experiences:**

1. Have you ever felt sad or had depressive symptoms for some time?
2. How long have you experienced sadness or depressive symptoms??
3. Has it been ongoing over time or just happened for short periods?

**For the next questions, let’s only consider your experiences with depression and the factors that may be associated with your depressive symptoms.**

| **Topic** | **Main questions** | **Probing questions according to the BPS factors** |
| --- | --- | --- |
| **Social domain** | 1. Is there something related to the background that causes you to feel sad or depressed? | - 1. Can you tell me more about… - 2. I’m not sure about that, can you give me an example? - 3. You have told me that you…can you explain more? - 4. You seem like (change of expression)…can you tell me what you feel right now? - 5. What do you have in your mind when you think about…? - 6. I heard you saying…..is that true from what I understand? - 7. Please correct me if I am wrong |
| **Psychological domain** | 1. Can you tell me about your experience of being stigmatized, if any? And how does it relate to your depressive symptoms? |  |
|  | 1. Can you tell me about the support that you receives from your surrounding? And how does it relate to your depressive symptoms? |  |
|  | 1. Is keeping your HIV status contribute to your depressive symptoms? |  |
|  | 1. Did your sexual relationship contribute to your depressive symptoms? |  |
|  | 1. Can you tell me about your experience with substance use? And how does it relate to your depressive symptoms? |  |
| **Biological domain** | 1. Can you tell me about your HIV treatment and how it affects your depressive symptoms? |  |
|  | 1. Is there something related to your health condition or other diseases that you think might contribute to your depressive symptoms? |  |

**Ending question**

1. Do you have anything further to add concerning my questions thus far??
2. Do you have any questions about this session and how the information will be used?

**Interview summary and validation**

Before I end this interview, I would like to summarize our conversation

(To perform a quick 5 minutes summary based on the researcher’s notes)

**Do you agree with the summary of our conversation today?**

Is there anything you want to add or correct?

**Conclusion**

Thank you for participating in this study. The information you provide is invaluable for this study and might help improve services for HIV-positive MSM. We will write a scientific report based on our conversation today and the previous survey you have answered before. I’d want to remind you that your personal information will be removed, and nobody will identify you from providing me with such information. If you have any questions regarding this study in the future, you may contact me anytime. Thank you very much, sir.
