# Supplementary material for: Biopsychosocial approach to understanding predictors of depressive symptoms among men who have sex with men living with HIV in Selangor, Malaysia: A mixed methods study protocol
Source: PLoS One. 2023 Jun 2;18(6):e0286816. doi: 10.1371/journal.pone.0286816 (PMC10237489; doi:10.1371/journal.pone.0286816)
Supplement: Attachment — Submitted filename: Rebuttal letter MSM protocol.docx [file pone.0286816.s002.docx]

Dec 31, 2022

Rebuttal Letter to manuscript:

**Biopsychosocial approach to understanding predictors of depressive symptoms among men who have sex with men living with HIV in Selangor, Malaysia: A mixed-methods study protocol**

Zul Aizat Mohamad Fisal^1^, Ahmad Zaid Fattah Azman^2^, Gurpreet Kaur^3^, Rosliza Abdul Manaf^2*^

^1^Faculty of Medicine and Health Sciences, University Putra Malaysia

^2^Department of Community Health Sciences, Faculty of Medicine and Health Sciences, University Putra Malaysia

^3^Klang District Health Office, Selangor, Malaysia

Dear Academic Editor and reviewer,

Thank you for your comments and recommendations, which allowed us to improve the paper. In the updated manuscript, we expect to answer all the issues identified. In this document, we answer all the questions the reviewers highlighted.

Comments are shown in bold font, followed by our answer/comment in normal font. The corrections/changes in the manuscript are displayed through the track changes.

**Reviewer #1:**

**1. ABSTRACT
- Abstract should be clearer in objectives that this is only a published protocol and not the results from the study that will be conducted in the future
- Add “%” to depression prevalence estimates**

Response:

-Dear reviewer, to clarify, the sentences were added as suggested: This is merely a published protocol, not the findings of a future study.

-% added to prevalence estimates. **2.BACKGROUND
- Is the prevalence for depression (43% t 51%) for a region, country or globally?**

**-** Dear reviewer, the prevalence was based on the previous systematic review and study (sentences added).

**- Page 4, define ART as it appears for first time**

**-** Dear reviewer, ART defined as antiretroviral therapy (ART)

**- Replace HIV-positive individual with “****individuals living with HIV”**

**-** Dear reviewer, the sentence was replaced as suggested.

**- Recommend adding more background statistics like the size of the study population in Malaysia to demonstrate public health impact. Can also give the proportions of Malay MSM compared to other Malay people living with HIV.**

**-** Dear reviewer, the prevalence of MSM population in Malaysia and MSM living with HIV compared to the others key populations has been added.

**- Is there any theoretical framework being used to generate the research questions and also to help guide instrument development for both phases of research?**

**-** Dear reviewer, the sentences added: “The study will use the biopsychosocial model as the theoretical framework to guide quantitative and qualitative phases. This framework incorporates biological, psychological, and social aspects to explain the intricacies and evolution of human behavior”.

**3.METHODS
- Pg. 7, end of bullet 3, missing word “factors” at the end of the statement**

**-** Word added.

**- Last sentence on page seven written in past tense so there is tense discord**

-The sentence was change to future tense.

**- Pg 8, should it be philosophy framework?**

**-**Yes, it should be philosophy framework.

**- Pg 8, what if all the sample or most of the sample in the quantitative phase has moderate or severe depression, how will the 20 participants for the qualitative phase be selected?**

**-** However, suppose all the respondents or most respondents in the quantitative phase have moderate or severe depression. In that case, those with severe depression will be chosen first, followed by moderately severe depression and moderate depression. The justification for this method is that the quantitative and qualitative data and their analysis might clarify and explain the statistical results through a more in-depth examination of the respondents’ perspectives.

**- Pg. 9/10 – are there eligibility criteria related to HIV status?**

**-** Yes, eligibility is MSM living with HIV. Sentences were corrected.

**- Pg. 10, is the questionnaire computer administered or otherwise?**

**-** Dear reviewer, a self-administered paper-based questionnaire will be used for this study to reduce non-response rates, as seen in another study.

**- Will an interview guide be developed for the in-depth interviews**

- Yes, the interview guide will be developed. The sentences were added under the Qualitative data collection method and process.

**- Logistic regression is proposed for analysis, are all the outcome variables dichotomous?**

**-** Dear reviewer , the binary outcomes of depressive symptoms will be using a cut-off score of 10. The scores of 0 to 9 represent none to subthreshold depression, and a score of 10 and above represent a spectrum of a patient to possible major depression based on depressive symptoms.

**- Is there any plan for missing variables or outliers?**

**-** Dear reviewer, all missing data will be assigned with missing value codes. The rules are: 1) missing value codes must be of the same data type as the data they present (e.g: for missing numeric data value codes must also be numeric; 2) Missing value codes cannot occur as data in the data set, and 3) By convention, the choice of the digit is -99.

**-**Any outliers will not be removed first. The simple approach is to analyze with outliers and without outliers and compare the results. If the results are similar, the outlier does not significantly influence the result. If the results change drastically, we will use the median (inter-quartile range) or 5% trimmed mean for descriptive results. The mean of the middle 90% of the observation will be taken by removing 5% of the upper and lower data. For the statistical test, we will carry out data transformation to avoid the result being affected by the outliers.

**- Recommend having more than one coder for the qualitative data analysis**

**-** Dear reviewer, thank you for the recommendation. Sentences added “ There will be three coders during the process. Multiple coders produce coding consistency or intercoder reliability by including two or more coders in the coding process, hence enhancing coding reliability”.

Dear Editors

We thank the reviewer for their generous comments on the manuscript and have edited it to address all the concerns.

We believe that the manuscript is now suitable for publication in Plos One.

Sincerely

Zul Aizat Mohamad Fisal

Doctor of Public Health candidate,

Faculty of Medicine & Health Sciences,

Universiti Putra Malaysia

On behalf of all authors.
