# Supplementary material for: Biopsychosocial approach to understanding predictors of depressive symptoms among men who have sex with men living with HIV in Selangor, Malaysia: A mixed methods study protocol
Source: PLoS One. 2023 Jun 2;18(6):e0286816. doi: 10.1371/journal.pone.0286816 (PMC10237489; doi:10.1371/journal.pone.0286816)
Supplement: Attachment — Submitted filename: Rebuttal 2 letter MSM protocol_ros.docx [file pone.0286816.s003.docx]

April 20, 2023

Rebuttal Letter to manuscript:

**Biopsychosocial approach to understanding predictors of depressive symptoms among men who have sex with men living with HIV in Selangor, Malaysia: A mixed-methods study protocol**

Zul Aizat Mohamad Fisal^1^, Rosliza Abdul Manaf^2*^, Ahmad Zaid Fattah Azman^2^, Gurpreet Kaur^3^

^1^Faculty of Medicine and Health Sciences, University Putra Malaysia

^2^Department of Community Health Sciences, Faculty of Medicine and Health Sciences, University Putra Malaysia

^3^Klang District Health Office, Selangor, Malaysia

Dear Academic Editor and reviewers,

Thank you for your comments and recommendations, which allowed us to improve the paper. In the updated manuscript, we have tried to improve all the issues identified. In this document, we answer all the questions the reviewers highlighted.

Reviewers comments are shown in bold font, followed by our answer/comment in normal font. The corrections/changes in the manuscript are displayed through the tracked changes.

**Reviewer #2:**

**Abstract
1. The background could use further attention to the socioecological factors mentioned that affect people living with HIV. It isn’t enough to state that these factors exist – how do they related specifically to the psychological wellbeing of people living with HIV?**
Thank you for the comments. We have corrected the background, but instead of using the word ‘socioecological factors’, we use biopsychosocial factors to link it with the title and the whole protocol.

**Introduction
1. The first paragraph would greatly benefit from varied sentence structure so that most sentences do not begin with “Depression”.**

The sentence structure has been rephrased and modified to ensure fluency and clarity.

**2. MSM should be defined prior to use of the abbreviation, as should HIV.**

The abbreviations have been defined.

**3. You should state the prevalence of depression among MSM to further make the point that depression prevalence is elevated among MSM with HIV.**

The prevalence of MSM has been stated, followed by the prevalence of MSM living with HIV for comparison.

**4. “MSM is likely to be the main key population in Malaysia by 2030.” I don’t think this says what you mean for it to say. Do you mean that “By 2030, MSM are projected to account for the largest share of prevalent HIV infections in Malaysia”**

Thank you for the suggestion. The sentence has been corrected as suggested.

**5. You do not sufficiently set up how “mental health information from MSM living with HIV” will be used. What value does this information bring? Does it inform interventions?**

The paragraph has been corrected with an addition on the value of mental health information in paragraph 6.

**6. Respectfully, I do not follow the decision to end the introduction by saying that this work will identify gaps in health services. You set up the protocol by wanting to understand factors that are associated with depression among MSM, and then discuss how this is of relevance because depression has been associated with non-adherence to HIV medications and other sexually risky behaviors. I would just make it clear that you’re focused on individual determinants of behavior – your data is not going to speak to gaps in health services because, from what I can tell, you are not proposing to assess these factors.**

The sentence on gaps in health services has been removed and replaced with the suggested correction.

**Methods
1. I am alarmed at the last sentence of the first paragraph in the methods: “The respondents will be given access to personal information… upon request”. Please clarify the meaning of this.**

The sentence has been changed and with reference for clarity. New sentence: “As part of the rights of the respondents, they are permitted access to personal data obtained concerning them.”

**2. The authors explain the phases and rationale for such phases, of their mixed-method design, well. Yet no attention is paid to data integration.**

A paragraph on data integration has been added in the methodology section. The part on data integration in detail was added in the discussion section as well.

**3. I do not follow the sample size justification. A sample size of nearly 1000 MSM with HIV to recruit in a period of approximately 4 months, even across 10 sites with an established sampling frame, seems improbable. Might you be able to speak to the feasibility of this based on past work by your team and or additional information about the recruitment sites? I also do not understand why the sample size calculation is not grounded in your statistical approaches.**

Dear editor, we wish to make a correction on that matter. The quantitative data collection started in March 2022 to February 2023 (IRB approval was in January 2022 as attached). The sample size calculation has been grounded in statistical approaches.

**4. If you are interested in an outcome of depression, why are you not screening participants to enroll those with clinically significant depressive symptoms? There is no guarantee that you will result in a sample of MSM with HIV who also have depression that is large enough to facilitate your analysis**

Depending on the research question and study design, it is more appropriate to include all HIV MSM regardless of their level of depressive symptoms. For example, if the goal of the study is to investigate the prevalence of depression among MSM living with HIV and its impact on HIV-related health outcomes, it would be important to enroll a representative sample of MSM living with HIV with varying levels of depressive symptoms. This would allow for prevalence to be measured and a more comprehensive understanding of the relationship between depression and HIV-related health outcomes in this population. However, it is debatable, and we fully respect your insight.

**5. Inclusion criteria – “Men identified as homosexual based on clinic database”. This will exclude MSM who are not homosexual. MSM is a behavior, not an identity.**

We have corrected the sentence. It would not impact the samples since the HIV databases in the respective clinics were based on self-identified men who have sex with men.

**6. It seems that site selection is conflated with participant selection. For example – criterion 4 is that there must be more than 200 MSM with HIV. This isn’t a participant level factor.**

Dear editor, criterion 4 has been removed, we only stated in the method section, that study location will be in the top ten clinics with the highest number of MSM living with HIV. This action also not affecting the sampling and sample size as the study location is still the same.

**7. No where does it say that the participants must have HIV in the inclusion criteria**

Thank you for the important point. We have added the criteria.

**8. Section 4 social support. PLHIV is used and not defined**

Thank you. All the abbreviations have been defined.

**9. Might you consider measuring other likely relevant constructs including: self-efficacy in adhering to medications, internalized MSM related stigma**

Thank you for the suggestion. For internalized stigma part of it has been grounded in the subscale of the 12-item short stigma scale. For self-efficacy and adherence to medication, we will consider it for the next research.

**10. It is not clear how random sampling will occur.**

In the quantitative arm of the study, proportionate random sampling was applied. The HIV databases from each clinic were obtained from the Selangor State Health Department to look for the list and the total number of patients in each clinic. Then, the required number of respondents in each clinic was calculated based on proportion to get 941 respondents. Based on the screened name list of MSM in each clinic, random sampling is conducted to select the required number of respondents using a randomizer application.

**11. It is not clear why you would need to do a pilot study with 30 people to look for internal consistency of the select items. Have these measures not performed well in other samples? I could see you wanting to pilot if you were creating original measures.**

Thank you. We totally agree with the statement. We have decided to remove the internal consistency conduct and only stick with the face validity and content validity.

**12. The section on the ethical reasons of linking people who screen with depression to care should be included prior to the section on analysis.**

Thank you. We agreed and followed the suggestion**.**

**13. You have not written about exploring the nature of the missing data nor considered imputation to address missingness, which you could do with a sample size as large as you have.**

We have updated the paragraph on handling missing data, followed the suggestion about exploring the nature of the missing data, and considered imputation to address missingness.

**14. With a proposed sample size as large as you have and for a research question like you pose, it is a missed opportunity to not engage in structural equation modeling. You’re really interested in a lot of latent variables that are challenging to conceptualize and measure that affect the experience of depression in an individual who is MSM and also HIV+**

Thank you for the suggestion. We will consider this in the next phase of the study.

**15. Despite this being a mixed methods study, no attention is paid to the integration of the data.**

Thank you for the very important input. We have explained the integration in the method and discussion section. The integration occurs in all research stages: study design, method, and report writing.

**Discussion
1. I find it problematic to describe recall measures as buffering against the limitation of cross-sectional data. The data is still cross-sectional in nature. Perhaps instead of stating this, you could reiterate the value of the cross-sectional data (point prevalence of depression among a large sample of MSM is very useful, understanding of factors associated with depression informs intervention, or identifying factors that are protective against depression is useful too!)**

Dear editor we totally agrred and have corrected the paragraph as suggested**.**

**2. Overall – it is less important to be discussing how withdrawal of participants will be handled. I would save that for unpublished material. What would be most informative is a discussion of how this data will be integrated (quant/qual mixing), reiteration of the relevance of this work. ETC.**

Thank you. We have removed the withdrawal part. We also have put a discsussion on the integration.

Dear Editors

Thank you again for your time and careful consideration of our manuscript. We appreciate the opportunity to respond to the reviewers' comments and hope we have addressed your concerns satisfactorily. We remain confident in the importance and validity of our findings, and we look forward to the possibility of publishing our work in your esteemed journal.

Sincerely

Zul Aizat Mohamad Fisal

Doctor of Public Health candidate,

Faculty of Medicine & Health Sciences,

Universiti Putra Malaysia

On behalf of all authors.
