# Supplementary material for: Biopsychosocial approach to understanding predictors of depressive symptoms among men who have sex with men living with HIV in Selangor, Malaysia: A mixed methods study protocol
Source: PLoS One. 2023 Jun 2;18(6):e0286816. doi: 10.1371/journal.pone.0286816 (PMC10237489; doi:10.1371/journal.pone.0286816)
Supplement: Attachment — Submitted filename: PONE-D-22-23739_R2.docx [file pone.0286816.s004.docx]

Please add line numbers for easy review.

**Abstract**

Conclusion: toward Ending AIDS in 2030 🡪 toward ending the AIDS epidemic by 2030

**Introduction**

1. Depression symptoms 🡪 Depressive symptoms

Please use depressive symptoms throughout the text.

2. ending AIDS 2023 🡪 ending the AIDS epidemic by 2030
3. Hypothesis: Your outcome variable is depressive symptoms. To present association, it is better to show the direction from exposure to the outcome, not the other way around. A (exposure) 🡪 B (outcome).

There is a significant association between depressive symptoms among MSM living with HIV and social factors 🡪 There is a significant association between social factors and depressive symptoms among MSM living with HIV.

**Methods**

1. Specific objective 3: Please reverse accordingly. Same comment as above.
2. Please improve the quality of figure 1. Include footnotes for abbreviations.
3. Avoid using the term “respondents” once you have already identified your respondents. It is better to specify than using general terms. Please update throughout the text.
4. Page 15: for further management 🡪 for further assessment

Results from PHQ-9 will require clinical assessment.

5. Page 16: Do you have a tentative semi-structured topic guide? It will be helpful if you will include this as a supplementary file.
6. Page 18: Please justify and cite your reference for choosing p-value <0.25 as criterion for the choice of variables to be included in multiple logistic regression model.
7. Page 24: Safety consideration 🡪 Safety considerations

**References**

1. Please follow PLOS ONES’ citation style, both in-text and reference list.
