# Supplementary material for: Biopsychosocial approach to understanding predictors of depressive symptoms among men who have sex with men living with HIV in Selangor, Malaysia: A mixed methods study protocol
Source: PLoS One. 2023 Jun 2;18(6):e0286816. doi: 10.1371/journal.pone.0286816 (PMC10237489; doi:10.1371/journal.pone.0286816)
Supplement: Attachment — Submitted filename: Rebuttal 2 MSM 5.5.23.docx [file pone.0286816.s005.docx]

May 8, 2023

Rebuttal Letter to manuscript:

**Biopsychosocial approach to understanding predictors of depressive symptoms among men who have sex with men living with HIV in Selangor, Malaysia: A mixed-methods study protocol**

Zul Aizat Mohamad Fisal^1^, Rosliza Abdul Manaf^2*^, Ahmad Zaid Fattah Azman^2^, Gurpreet Kaur^3^

^1^Faculty of Medicine and Health Sciences, University Putra Malaysia

^2^Department of Community Health Sciences, Faculty of Medicine and Health Sciences, University Putra Malaysia

^3^Klang District Health Office, Selangor, Malaysia

Dear Academic Editor and reviewers,

Thank you again for your comments and recommendations, which allowed us to improve the paper. We have tried to improve all the issues identified in the updated manuscript. In this document, we answer all the questions the reviewers highlighted.

Reviewers comments are shown in bold font, followed by our answer/comment in normal font. The corrections/changes in the manuscript are displayed through the tracked changes.

Comment and answer

**Please add line numbers for easy review.**

Line has been added

**Abstract**

**Conclusion: toward Ending AIDS in 2030  toward ending the AIDS epidemic by 2030**

We have corrected the sentences throughout the manuscript.

**Introduction**

**1. Depression symptoms  Depressive symptoms**

**Please use depressive symptoms throughout the text.**

We have corrected the sentences throughout the manuscript.

**2. ending AIDS 2023  ending the AIDS epidemic by 2030**

We have corrected the sentences throughout the manuscript.

**3. Hypothesis: Your outcome variable is depressive symptoms. To present association, it**

**is better to show the direction from exposure to the outcome, not the other way**

**around. A (exposure)  B (outcome).**

**There is a significant association between depressive symptoms among MSM living**

**with HIV and social factors  There is a significant association between social**

**factors and depressive symptoms among MSM living with HIV.**

We have corrected the sentences for the hypotheses.

**Methods**

**1. Specific objective 3: Please reverse accordingly. Same comment as above.**

We have corrected the sentences

**2. Please improve the quality of figure 1. Include footnotes for abbreviations.**

The figure has been improved to make it self-explanatory, abbreviations have been added, and figure has been converted using PACE.

**3. Avoid using the term “respondents” once you have already identified your**

**respondents. It is better to specify than using general terms. Please update throughout**

**the text.**

The “respondents” term has been changed to “MSM” as it is commonly used in academic research related to sexual health and behavior among men who have sex with men.

**4. Page 15: for further management  for further assessment**

**Results from PHQ-9 will require clinical assessment.**

The sentence has been changed as suggested.

**5. Page 16: Do you have a tentative semi-structured topic guide? It will be helpful if you**

**will include this as a supplementary file.**

Sure. The interview protocol was attached as a supplementary file.

**6. Page 18: Please justify and cite your reference for choosing p-value &lt;0.25 as criterion for the choice of variables to be included in multiple logistic regression model.**

The reason has been justified with the sentences: A less rigorous threshold, such as a P-value < 0.25, should be applied to prevent omitting crucial variables from a model due to stochastic variation [38,39]. Therefore, variables with a P value < 0.25 will be included in multiple logistic regression to measure the predictors of depressive symptoms.

**7. Page 24: Safety consideration  Safety considerations**

We followed the suggestion**.**

**References**

**1. Please follow PLOS ONES’ citation style, both in-text and reference list.**

The citation has been adjusted to follow the Vancouver and PLOS ONES’ style. Duplicate citation were removed.

Dear Editors

Thank you again for your time and careful consideration of our manuscript. We appreciate the opportunity to respond to your comments and hope we have addressed your concerns satisfactorily. If any problems arise, we are ready to improve the manuscript again. We remain confident in the importance and validity of our findings, and we look forward to the possibility of publishing our work in your esteemed journal.

Sincerely

Zul Aizat Mohamad Fisal

Doctor of Public Health candidate,

Faculty of Medicine & Health Sciences,

Universiti Putra Malaysia

On behalf of all authors.
